# Evidence of differential spreading events of grapevine pinot Gris virus in Italy using datamining as a tool

**DOI:** 10.1007/s10658-021-02343-3

**Published:** 2021-08-26

**Authors:** Jean-Michel Hily, Véronique Komar, Nils Poulicard, Amandine Velt, Lauriane Renault, Pierre Mustin, Emmanuelle Vigne, Anne-Sophie Spilmont, Olivier Lemaire

**Affiliations:** 1 IFV, Le Grau-Du-Roi, France; 2 Université de Strasbourg, INRAE, SVQV UMR-A 1131, F-68000 Colmar, France; 3 PHIM Plant Health Institute, Univ Montpellier, IRD, CIRAD, INRAE, Institut Agro, Montpellier, France

**Keywords:** Grapevine, GPGV, Detection, Datamining

## Abstract

Since its identification in 2003, grapevine Pinot gris virus (GPGV, *Trichovirus*) has now been detected in most grape-growing countries. So far, little is known about the epidemiology of this newly emerging virus. In this work, we used datamining as a tool to monitor *in-silico* the sanitary status of three vineyards in Italy. All data used in the study were recovered from a work that was already published and for which data were publicly available as SRA (Sequence Read Archive, NCBI) files. While incomplete, knowledge gathered from this work was still important, with evidence of differential accumulation of the virus in grapevine according to year, location, and variety-rootstock association. Additional data regarding GPGV genetic diversity were collected. Some advantages and pitfalls of datamining are discussed.

Since its characterization in Italy (Giampetruzzi et al., 2012), grapevine Pinot gris virus (GPGV, *Trichovirus*, *Betaflexiviridae*) has been detected in most grapevine growing regions around the world. Generally, the virus is detected using serological and/or molecular tools. In this work, we describe datamining as a potential additional method to identify grapevine infected with this virus, better estimating its distribution worldwide. While this specific work cannot be considered as an epidemiological study per se, it still unquestionably offers valuable information on the virus (i.e.*,* its geographic distribution and genetic composition), providing a snapshot of the situation in three different vineyards in Italy at a specific time, giving new insight on GPGV accumulation, introduction and transmission.

This particular work is based on the data provided by a study on the contribution of genotype, the environment and their interaction to the berry transcriptome that was previously published (Dal Santo et al., 2018). Two cultivars, Cabernet Sauvignon and Sangiovese, were planted in three different locations: Montalcino, Bolgheri and Riccione. The former two Italian cities are located in the Tuscany hills and Tuscany coast respectively, while the latter is positioned on the Adriatic coast (Fig. 1). To minimize genetic variation, researchers used the same clonal material for each cultivar, with clones R5 and VCR23 of Cabernet Sauvignon and Sangiovese, respectively. In addition, three different rootstocks were tested in the study: Kober-5BB, 420A and 161.49 C. After uploading the 72 SRA files generated from this work, all samples were analyzed for the presence of GPGV using Workbench 12.0 software (CLC Genomics Workbench, Aarhus, Denmark) as previously described (Hily et al., 2018). This was first assessed by mapping reads to a collection of curated GPGV reference sequences. For those displaying reads corresponding to GPGV, de novo assembly steps were performed and further extended by multiple rounds of residual reads mapping as previously described (Nourinejhad Zarghani et al., 2018). Genome sequences being produced were ascertained using very stringent mapping parameters (length of 0.95/similarity of 0.95).

**Fig. 1:**
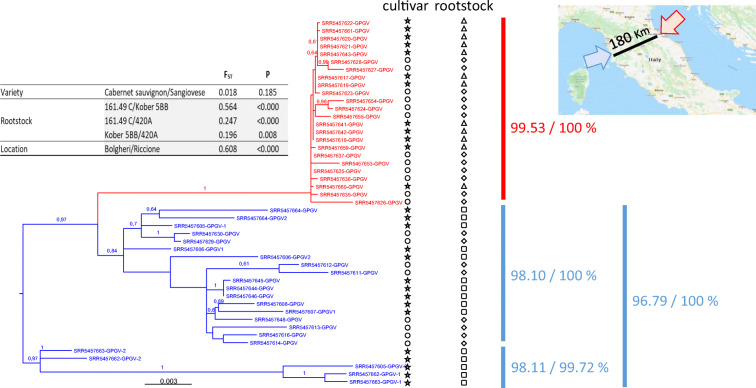
Maximum-likelyhood tree inferred from sequences (7206 nt) of grapevine Pinot gris virus genome isolated from two cultivars, Cabernet Sauvignon clone R5 (star) and Sangiovese clone VCR23 (circle). Rootstocks are also indicated with 161.49 C (square), Kober 5BB (triangle) and 420A (diamond). Only bootstraps above 0,5 are shown. Colors correspond to the location in Italy where samples were recovered, Bolgheri (blue) and Riccione (red), see map on the upper right corner. Identity percentages between sequences are indicated on the right of the ML-tree. Measurements of population’s differentiation (fixation index, F_ST_) and associated statistics (*P* value) are on the upper left corner

Our datamining study revealed that only samples from Bolgheri and Riccione were positive for GPGV. The virus was hardly detected in a few samples from Montalcino (Table 1); however, no complete sequence could be recovered. These ‘Low Read Count’ samples were probably the result of ‘intra-lane contamination’, as previously described in other studies (Vigne et al., 2018). When using RPKM (Reads per kilo base per million) data as a proxi for virus accumulation in the samples, our analyses revealed differential accumulation of GPGV according to many variables (Fig. 2). Indeed, GPGV seems to accumulate more in berries in 2011 than in 2012 (*P* < 10^−5^) and at a later stage of fruit development, at mid-ripening rather than pre-veraison (P < 10^−4^). Also, the association cultivar-rootstock seems to have its importance in virus accumulation. Indeed, GPGV seems to accumulate more in Cabernet Sauvignon cultivar grafted onto either 161–49 or Kober-5BB rootstocks, rather than in Sangiovese grafted onto 420A at either location (*P* ≤ 10^−4^). In addition, differential accumulation of GPGV was also observed according to location where grapevines were grown (P < 10^−5^), with GPGV accumulating more in Riccione than in Bolgheri.

**Table 1:** All information regarding the datamining analyses performed from the study from Dal Santo et al., 2018

SEA #	hybridization #	Sample ID	Cultivar	Rootstock	Developmental Stage	Location	Vintage	GPGV	RPKM	Mapped read counts*	Total read counts	Genome length (nt)
SRR5457593	4	CS_MO_PV_11_A	Cabernet Sauvignon	S04	Pre-veraison	Montalcino	2011				39,659,627	
SRR5457594	5	CS_MO_PV_11_B	Cabernet Sauvignon	S04	Pre-veraison	Montalcino	2011				37,953,191	
SRR5457595	6	CS_MO_PV_11_C	Cabernet Sauvignon	S04	Pre-veraison	Montalcino	2011				45,920,500	
SRR5457596	7	CS_MO_MR_11_A	Cabernet Sauvignon	S04	Mid-ripening	Montalcino	2011				30,131,817	
SRR5457597	8	CS_MO_MR_11_B	Cabernet Sauvignon	S04	Mid-ripening	Montalcino	2011				25,466,144	
SRR.5457598	9	CS_MO_MR_11_C	Cabernet Sauvignon	S04	Mid-ripening	Montalcino	2011				29,627,432	
SRR5457599	16	SG_MO_PV_11_A	Sangiovese	420A	Pre-veraison	Montalcino	2011				30,253,594	
SRR5457600	17	SG _MO_PV_11_B	Sangiovese	420A	Pre-veraison	Montalcino	2011				27,619,510	
SRR5457601	18	SG _MO_PV_11_C	Sangiovese	420A	Pre-veraison	Montalcino	2011				24,825,638	
SRR5457602	19	SG _MO_MR_11_A	Sangiovese	420A	Mid-ripening	Montalcino	2011				31,261,949	
SRR5457603	20	SG _MO_MR_11_B	Sangiovese	420A	Mid-ripening	Montalcino	2011				37,850.541	
SRR5457604	21	SG _MO_MR_11_C	Sangiovese	420A	Mid-ripening	Montalcino	2011				33,319,419	
SRR5457605	28	CS_BO_PV_11_A	Cabernet Sauvignon	161–49	Pre-veraison	Polgheri	2011	2	91,43	19,934	30,211,399	7287, 7287
SRR5457606	29	CS_BO_PV_11_B	Cabernet Sauvignon	161–49	Pre-veraison	Polgheri	2011	2	44,32	10,113	31,519,652	7254, 7254
SRR5457607	30	CS_BO_PV_11_C	Cabernet Sauvignon	161–49	Pre-veraison	Polgheri	2011	1	100,96	25,001	34,310,824	7247
SRR5457605	31	CS_BO_MR_11_A	Cabernet Sauvignon	161–49	Mid-ripening	Polgheri	2011	1	173,45	42,993	34,345,114	7247
SRR5457609	32	CS_BO_MR_11_B	Cabernet Sauvignon	161–49	Mid-ripening	Polgheri	2011				32,004,939	
SRR5457610	33	CS_BO_MR_11_C	Cabernet Sauvignon	161–49	Mid-ripening	Polgheri	2011				32,253,343	
SRR5457611	40	SG_BO_PV_11_A	Sangiovese	420A	Pre-veraison	Polgheri	2011	1	10,43	2425	32,216,454	7243
SRR5457612	41	SG_BO_PV_11_B	Sangiovese	420A	Pre-veraison	Polgheri	2011	1	9,64	2092	30,065,198	7240
SRR5457613	42	SG_BO_PV_11_C	Sangiovese	420A	Pre-veraison	Polgheri	2011	1	4,52	922	25,270,284	7213
SRR5457614	43	SG_BO_MR_11_A	Sangiovese	420A	Mid-ripening	Polgheri	2011	1	24,92	6360	35,361,602	7307
SRR5457615	44	SG_BO_MR_11_B	Sangiovese	420A	Mid-ripening	Polgheri	2011				30,185,292	
SRR5457616	45	SG_BO_MR_11_C	Sangiovese	420A	Mid-ripening	Polgheri	2011	1	68,12	15.589	31,708,932	7290
SRR5457617	52	CS_RI_PV_11_A	Cabernet Sauvignon	Kober-5BB	Pre-veraison	Riccione	2011	1	128,03	28,440	30,778,512	7254
SRR5457618	53	CS_RI_PV_11_B	Cabernet Sauvignon	Kober-5BB	Pre-veraison	Riccione	2011	1	128,00	27,080	29,314,935	72.54
SRR5457619	54	CS_RI_PV_11_C	Cabernet Sauvignon	Kober-5BB	Pre-veraison	Riccione	2011	1	111,44	28,416	35,330,755	7254
SRR5457620	55	CS_RI_MR_11_A	Cabernet Sauvignon	Kober-5BB	Mid-ripening	Riccione	2011	1	2258,35	48.5449	29,784,834	7254
SRR5457621	56	CS_RI_MR_11_B	Cabernet Sauvignon	Kober-5BB	Mid-ripening	Riccione	2011	1	2225,20	455,935	28,390,737	72.54
SRR5457622	57	CS_RI_MR_11_C	Cabernet Sauvignon	Kober-5BB	Mid-ripening	Riccione	2011	1	1565,76	284,493	25,176,180	72.54
SRR5457623	64	SG_RI_PV_11_A	Sangiovese	420A	Pre-veraison	Riccione	2011	1	89,48	17,871	27,673,291	7258
SRR5457624	65	SG_RI_PV_11_B	Sangiovese	420A	Pre-veraison	Riccione	2011	1	58,23	11,621	27,651,896	7254
SRR5457625	66	SG_RI_PV_11_C	Sangiovese	420A	Pre-veraison	Riccione	2011	1	135,88	27,403	27,943,588	7254
SRR5457626	67	SG_RI_MR_11_A	Sangiovese	420A	Mid-ripening	Riccione	2011	1	299,59	48,006	22,202,853	7289
SRR5457627	68	SG_RI_MR_11_B	Sangiovese	420A	Mid-ripening	Riccione	2011	1	414,33	89,289	29,860,068	7254
SRR5457628	59	SG_RI_MR_11_C	Sangiovese	420A	Mid-ripening	Riccione	2011	1	300,74	61,377	28,278,938	7254
SRR5457629	91	SG_BO_PV_12_A	Sangiovese	420A	Pre-veraison	Bolgheri	2012	1	3,99	884	30,685,737	7214
SRR5457630	92	SG_BO_PV_12_B	Sangiovese	420A	Pre-veraison	Bolgheri	2012	1	3,29	684	28,765,541	7250
SRR5457631	93	SG_BO_PV_12_C	Sangiovese	420A	Pre-veraison	Bolgheri	2012	✓	*1,87*	455	33,797,617	7131
SRR5457632	94	SG_MO_PV_12_A	Sangiovese	420A	Pre-veraison	Montalcino	2012	✓	*0,69*	143	28,565,019	4800
SRR5457633	95	SG_MO_PV_12_B	Sangiovese	420A	Pre-veraison	Montalcino	2012	✓	*2,99*	675	31,322,839	7201
SRR5457634	96	SG_MO_PV_12_C	Sangiovese	420A	Pre-veraison	Montalcino	2012	✓	*1.43*	312	30,193,456	6686
SRR5457635	97	SG_RI_PV_12_A	Sangiovese	420A	Pre-veraison	Riccione	2012	1	46,29	10,705	32,044,752	7257
SRR5457636	98	SG_RI_PV_12_B	Sangiovese	420A	Pre-veraison	Riccione	2012	1	41,83	7769	25,735,588	7253
SRR5457637	99	SG_RI_PV_12_C	Sangiovese	420A	Pre-veraison	Riccione	2012	1	38,53	8245	29,653,480	7271
SRR5457539	100	CS_MO_PV_12_A	Sangiovese	420A	Pre-veraison	Montalcino	2012				28,374,413	
SRR5457639	101	CS_MO_PV_12_B	Cabernet Sauvignon	S04	Pre-veraison	Montalcino	2012				39,038,471	
SRR5457640	102	CS_MO_PV_12_C	Cabernet Sauvignon	S04	Pre-veraison	Montalcino	2012				29,599,165	
SRR5457641	103	CS_RI_PV_12_A	Cabernet Sauvignon	Kober-5BB	Pre-veraison	Riccione	2012	1	55,19	10,488	26,329,353	7253
SRR5457642	104	CS_RI_PV_12_B	Cabernet Sauvignon	Kober-5BB	Pre-veraison	Riccione	2012	1	50,26	11,045	30,452,556	7253
SRR5457643	105	CS_RI_PV_12_C	Cabernet Sauvignon	Kober-5BB	Pre-veraison	Riccione	2012	1	63,52	14,937	32,582,117	7253
SRR5457644	106	CS_BO_PV_12_A	Cabernet Sauvignon	161–49	Pre-veraison	Bolgheri	2012	1	20,46	2295	15,541,092	7277
SRR5457645	107	CS_BO_PV_12_B	Cabernet Sauvignon	161–49	Pre-veraison	Bolgheri	2012	1	14,68	2996	28,275,962	7223
SRR5457646	108	CS_BO_PV_12_C	Cabernet Sauvignon	161–49	Pre-veraison	Bolgheri	2012	1	26,84	12,934	66,769,968	7282
SRR5457647	109	SG_BO_MR_12_A	Sangiovese	420A	Mid-ripening	Bolgheri	2012	✓	*0,97*	216	30,804,911	6126
SRR5457648	110	SG_BO_MR_12_B	Sangiovese	420A	Mid-ripening	Bolgheri	2012	1	6,31	1463	32,129,314	7219
SRR5457649	111	SG_BO_MR_12_C	Sangiovese	420A	Mid-ripening	Bolgheri	2012	✓	*2.15*	388	25,018,444	6948
SRR5457650	112	SG_MO_MR_12_A	Sangiovese	420A	Mid-ripening	Montalcino	2012				24,003,382	
SRR5457651	113	SG_MO_MR_12_B	Sangiovese	420A	Mid-ripening	Montalcino	2012				37,168,759	
SRR5457652	114	SG_MO_MR_12_C	Sangiovese	420A	Mid-ripening	Montalcino	2012				29,938,586	
SRR5457653	115	SG_RI_MR_12_A	Sangiovese	420A	Mid-ripening	Riccione	2012	1	28,48	7041	34,255,543	7250
SRR5457654	116	SG_RI_MR_12_B	Sangiovese	420A	Mid-ripening	Riccione	2012	1	31,09	6790	30,258,155	7250
SRR5457655	117	SG_RI_MR_12_C	Sangiovese	420A	Mid-ripening	Riccione	2012	1	7,48	1524	28,230,567	7255
SRR5457656	118	CS_MO_MR_12_A	Sangiovese	420A	Mid-ripening	Montalcino	2012	✓	*1,05*	224	29,549,033	6431
SRR5457657	119	CS_MO_MR_12_B	Cabernet Sauvignon	S04	Mid-ripen^−^ng	Montalcino	2012				22,749,636	
SRR5457658	120	CS_MO_MR_12_C	Cabernet Sauvignon	S04	Mid-ripening	Montalcino	2012				29,723,920	
SRR5457659	121	CS_RI_MR_12_A	Cabernet Sauvignon	Kober-5BB	Mid-ripening	Riccione	2012	1	408,02	82,399	27,982,523	7250
SRR5457660	122	CS_RI_MR_12_B	Cabernet Sauvignon	Kober-5BB	Mid-ripening	Riccione	2012	1	923,80	235,636	35,343,145	7284
SRR5457661	123	CS_RI_MR_12_C	Cabernet Sauvignon	Kober-5BB	Mid-ripening	Riccione	2012	1	1336,91	318,331	32,992,910	7277
SRR5457662	124	CS_BO_MR_12_A	Cabernet Sauvignon	161–49	Mid-ripening	Bolgheri	2012	2	117,96	24,447	28,741,196	7217,7217
SRR5457663	125	CS_130_MR_12_B	Cabernet Sauvignon	161–49	Mid-ripening	Bolgheri	2012	2	74,49	209 41	38.951034	7217,7217
SRR5457664	126	CS_130_MR_12_C	Cabernet Sauvignon	161–49	Mid-ripening	Bolgheri	2012	2	63,53	15,221	33,195,577	7217,7217

The ‘number’ in the GPGV column correspond to the number of complete genome assembled in de novo in each sample. ✓ indicates that reads have mapped onto GPGV genome, as shown in the Mapped read counts columns would indicate, however no complete genome from contiguous sequence could be obtained and RPKM (Read per Kilobase Million) were always below 3 when no genome were assembled. This work was performed using CLC-Workbench using very stringent mapping parameters * (0,95/0,95)

**Fig. 2:**
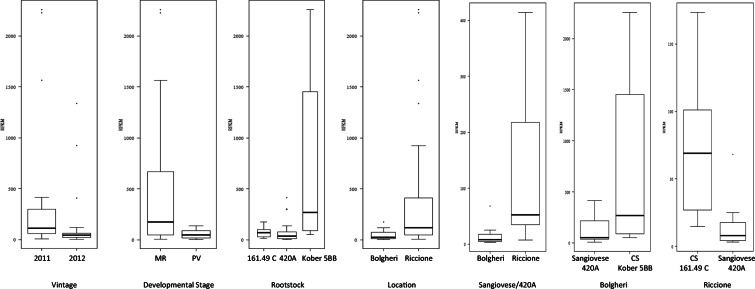
Box plot diagrams of RPKM in function of different variables. From left to right: year, developmental stage (MR: mid-ripening, PV: pre-veraison), rootstock, overall location, Sangiovese grafted onto 420A, grapevine cultivated in Bolgheri and in Riccione (CS: Cabernet Sauvignon). On each box, the central line is the median, the edges of the boxes are the 25th and 75th percentiles, the whiskers extend to the most extreme data and the dots refer to the outliers. Since RPKM values did not follow a normal distribution, a generalized linear model (GLM) with Poisson link function was used. The significance of the considered effect was tested using Wald chi2 test and the *p* values smaller than 0.05 threshold were considered statistically significant. All analyses and graphic representations were made with the R software version 4.0.2 (R core Team 2012)

When delving into the genetics of the virus, other information was revealed. Overall, 47 complete genome GPGV sequences (or near complete, covering at least all open reading frames) were assembled (Table 1), all submitted to GenBank (BK011089-BK011101, and the other sequences are available upon request). After a phylogenetic analysis (Fig. 1), three major clades of GPGV were found to infect these grapevines, each displaying a high intra-clade nucleic acid identity percentage ≥ 98.10%. Interestingly, GPGV sequences seemed to cluster together very well by location (Fig. 1, colors) however independently from cultivar. Fixation index (F_ST_) analyses (Fig. 1) confirmed the genetic differentiation of the viral population according to location, showing a statistically significant high F_ST_ value (F_ST_ = 0.608, *P* ≤ 10^−5^). Such segregation by location was also highlighted for grapevines grafted onto rootstock 161.49 C used exclusively in Bolgheri and grapevines onto Kober 5BB exclusively used in Riccione (F_ST_ = 0.564, P ≤ 10^−5^). Comparison of sequences obtained from the 420A rootstock also displayed statistically significant F_ST_ values; however, the values were lower than the ones mentioned above. This is most likely because 420A was used as a rootstock in both locations. Furthermore, the genetic background of the grapevine cultivar, which was also present in both locations, had no statistically significant impact on viral populations (F_ST_ = 0.018, *P* = 0.185).

In addition to the presence/absence of GPGV in the samples, this work highlights two distinct situations at the viral genomic level. Indeed, one vineyard is infected by a single variant, identity percentage ≥ 99%, as previously defined for GPGV (Hily et al., 2020), represented here by samples from the Bolgheri region, while the other vineyard (Riccione) is infected by at least two (or more) variants. These results indirectly, but strongly, suggest probable independent introduction/transmission events of GPGV in two out of the three locations specifically looked at, in this transcriptomic study. These situations are probably the result of transmission events through grafting (Saldarelli et al., 2014) and movement of infected material as previously suggested (Al Rwahnih et al., 2016; Fajardo et al., 2017; Wu & Habili, 2017). They may also have occurred horizontally by vectors either in the nursery or in the vineyard, with distinct variants of the virus being detected at each location, regardless of the clonal background of the grapevine. In addition, the detection of these different variants according to location, each displaying probable differences in fitness, may results in differential virus accumulation as observed above. Overall, this in silico work add onto the so-far limited knowledge on the natural transmission of GPGV in vineyards (Bertazzon et al., 2020; Hily et al., in press).

Lately, datamining is becoming a very important and powerful tool to identify new pathogens, as well as new variants of known viruses, such as from the now well-known *Coronaviridae* family for example (https://virological.org/t/serratus-the-ultra-deep-search-to-discover-novel-coronaviruses/516) (last visited 04/2021). Datamining can be also utilized to increase the number of complete genome sequences for downstream studies on the evolutionary history of specific viruses for example (Hily et al., 2020). In this work, datamining can be considered as an *in-silico* tool to monitor *post facto* the sanitary status of any vineyards around the world from which data have already been collected, published and made publicly available. There are a few pitfalls regarding datamining as a tool. Indeed, we do not have always all the details regarding the samples (i.e. metadata about the samples such as the exact origin and location of collection). We do not have the choice of the technology with which data were obtained nor the quality of the sample. However, the information being generated is still very valuable, it has already been paid for and therefore almost free (other than the time of analysis), it is available to anyone and most of all, it is ever growing.
